# Mandibular width as a novel anthropometric measure for assessing obstructive sleep apnea risk

**DOI:** 10.1097/MD.0000000000014040

**Published:** 2019-01-25

**Authors:** Hillel S. Maresky, Miriam M. Klar, Jaron Tepper, Haim Gavriel, Tomer Ziv Baran, Colin M. Shapiro, Sigal Tal

**Affiliations:** a Department of Radiology, Assaf Harofeh Medical Center, affiliated with Sackler Faculty of Medicine, Tel Aviv University, Tel-Aviv, Israel; b Hospital for Sick Children, Department of Medical Imaging, University of Toronto, Toronto, Ontaria, Canada; c Department of Otolaryngology, Assaf Harofeh Medical Center, Affiliated with Sackler Faculty of Medicine; d Department of Epidemiology and Preventive Medicine, School of Public Health, Sackler Faculty of Medicine, Tel Aviv University, Tel-Aviv, Israel; e Department of Psychiatry, Toronto Western Hospital, University Health Network, University of Toronto, Toronto, Ontario, Canada.

**Keywords:** craniofacial abnormality, mandible width, obstructive sleep apnea, sleep disordered breathing, STOP-BANG

## Abstract

Craniofacial abnormalities are a known obstructive sleep apnea (OSA) risk factor, but still need to be better characterized. This study investigates the relationship between mandibular width and the risk of developing OSA.

We retrospectively analyzed 3D reconstructions of head and neck computed tomography (CT) scans at our institution for mandibular width, neck circumference, neck fat volume (NFV), airway volume (AWV), and NFV:AWV ratio. Age, gender, and BMI were also documented. Patients were contacted to complete a STOP-BANG survey to assess OSA risk. Only patients with reconstructable scans and completed STOP-BANG questionnaires were included in the study. Survey results were analyzed to assess the correlation between mandible width and STOP-BANG. Mandible association was also compared to the associations of the other known risk factors.

The final analysis included 427 patients with a mean age of 58.98 years (standard deviation = 16.77), 56% of whom were male. Mandibular width was found to positively correlate with STOP-BANG score (r = .416, *P < *.001). Statistically significant differences between mandible size for each risk group was seen (*P < *.001). After controlling for age and sex, mandible size was significantly different only for the low risk vs. high risk groups (odds ratio = 1.11; 95% confidence interval = 1.03–1.20; *P = *.007). Furthermore, when stratified according to mandible size, the small mandible group (<77.50 mm) predominantly consisted of low risk patients; the medium size mandible group (77.50–84.40 mm) was predominated by intermediate risk patients, and large mandible (>84.40 mm) was predominantly seen in high risk patients. Mandible width expressed a stronger association than NFV:AWV ratio, but neck circumference and NFV had stronger associations than did mandible width.

In addition to previously documented OSA risk factors, mandibular width is positively correlated with OSA as an independent risk factor. Observation of a wide mandible (jaw) should raise awareness of OSA risk and increase screening methods when appropriate.

## 1 Introduction

Obstructive sleep apnea (OSA), characterized by frequent pauses in breathing due to airway closure during sleep,^[1,2]^ is a severe public health concern, affecting approximately 15% of the US population.^[1]^ OSA risk factors include male sex, increased age, elevated BMI, and anatomical abnormalities of the craniofacial region and upper airway.^[3–6]^ Research has indicated increasing prevalence^[3]^ with a prediction for continued increase due to an increase in prevalence of risk factors associated with OSA.^[7]^

While short-term effects include snoring and daytime sleepiness,^[1]^ long-term sequelae of this disorder are more severe. OSA is as a risk factor for cardiovascular diseases,^[1–4,7,8]^ such as hypertension and fluctuations in heart rate and blood pressure,^[3,9]^ ischemic heart disease, heart failure, atrial fibrillation,^[9]^ and stroke.^[1,7,8]^ It causes metabolic dysfunction^[4]^ and insulin resistance,^[9]^ hypercoagulable states,^[10]^ and impaired cognitive functioning.^[3,4,11]^ Impaired daytime behavioral functioning^[1]^ can lead to changes in mood and overall quality of life,^[3,7,8]^ including depression^[1,4,12]^ and anxiety.^[4]^ OSA can even lead to death, if severe enough.^[1–3,7,8]^ Proper treatment with continuous positive airway pressure (CPAP) can prevent long-term sequelae of OSA,^[2,7]^ although up to 80% of individuals who could benefit from treatment remain undiagnosed in the U.S.^[7,13]^

Previously, OSA research revolved around understanding OSA prevalence and age, obesity, and gender risk factors. Recently there has been a shift in OSA research towards focusing on the anatomic risk factors for OSA, in an attempt to better understand the OSA pathophysiology.^[9]^ It appears that patients with OSA have different craniofacial morphology compared to those without OSA.^[6]^ Of these craniofacial skeletal and soft tissue structure abnormalities, those most commonly associated with OSA include mandibular deficiency, maxillary hypoplasia, inferior position of hyoid bone, a narrow posterior air space, a greater flexion of the cranial base, elongation of the soft palate,^[6]^ and increased upper airway adipose tissue and reduction in the size of craniofacial structures.^[9]^ Even with these observations, additional categorization of these structures is needed.

Despite the abundance of research on craniofacial abnormalities implicated in OSA, literature on this topic excludes jaw width.^[6]^ Meanwhile, many alternative OSA treatments to CPAP revolve around the mandible, such as mandibular advancement devices, suggesting a connection between the mandible and presence of OSA. Therefore, our study aims to better analyze, understand, and describe some of these craniofacial abnormalities, most specifically mandible size, associated with OSA. Successful elucidation of these anatomic susceptibilities will produce significant clinical implications ranging from screening to therapeutic purposes.

## 2 Methods

### 2.1 Overview

The study was approved by the hospital's Institutional Review Board. We conducted a retrospective analysis on all head and neck CT scans, both with and without contrast injection, performed between January 2013 and December 2013 at our institution.

### 2.2 Participants

All 660 patients undergoing CT of the head and neck during the study time period, of all ages and for all indications, were considered for reconstruction. Exclusion criteria included patients below 18 years of age and scans not reconstructable for technical reasons, such as severe movement artifacts from breathing and intubated patients. All other CT scans were included in the study.

### 2.3 Study design & image analysis

Scans were performed on either a Philips Brilliance 64 CT scanner or a Philips Brilliance iCT 256 slice according to machine availability, with CT and computed tomography angiography (CTA) protocols divided between the 2 gantries based upon clinical indication for scan performance. CT protocol (256 MDCT protocol, and in brackets 64 MDCT, if different from 256 MDCT) was kVP 120, mAs 250 (220) with dose modulation, slice thickness 2 mm, increment 1.5 mm, rotation time 0.5 seconds, field of view 250 mm. CTA protocol (256 MDCT protocol, and in brackets 64 MDCT, if different from 256 MDCT) was kVP 120, mAs 300 with dose modulation, slice thickness 0.9 mm (1 mm), increment 0.45 mm (0.5 mm), rotation time 0.5 seconds, field of view 220 mm. A bolus tracking technique (automated tracking for enhancement of the aortic arch lumen during bolus contrast material injection) was included in the protocol.

Image postprocessing and 3D reconstruction was conducted by a single blinded radiologist (HSM) using Philips Intelligence software (v5.02.1001), a dedicated postprocessing software. Semiautomated caliper tools were used for all measurements.

**Figure 1:**
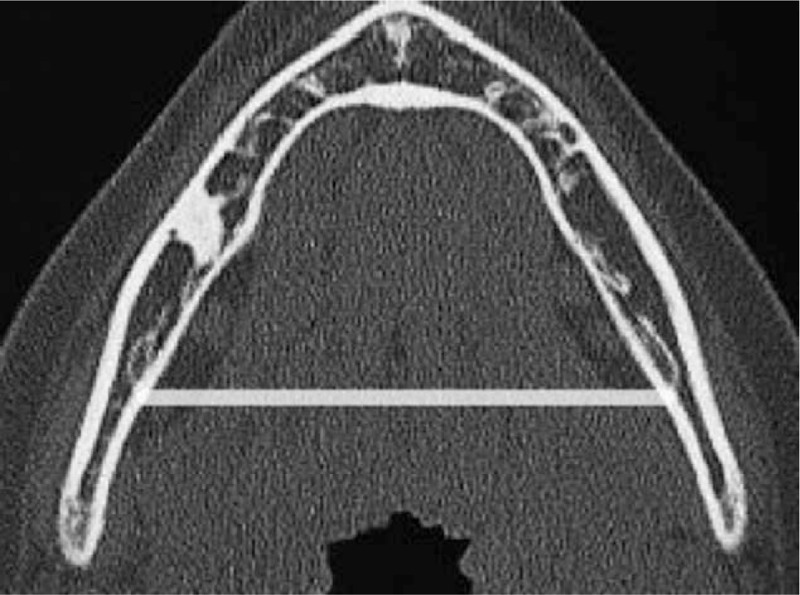
Axial measurement of inter-mandibular width via CT. CT = computed tomography.

Bony measurements of the mandible were made in the axial distance between the gonial angles (Fig. 1), and sagitally between the gonial angle and clivus, in millimeters. Neck fat volume (NFV) reconstruction was evaluated from the raw data from the hard palate superiorly to the sternal angle of Louis inferiorly. Airway volume (AWV) reconstruction was deduced from the raw data from the nasopharynx superiorly, to the first tracheal cartilage inferiorly. Validation studies on NFV and AWV volume calculations were performed on by a second reader, a separate blinded radiologist, using the same software on 20 random CT protocols and 20 random CTA protocols. Axial anthropometric measurements, mimicking the circumference of the neck, were acquired by calculating the surface area (in cubic millimeters), with slices perpendicular to the cervical spine at the level of the soft palate superiorly (axial level 1), and the thyroid cartilage inferiorly (axial level 2). Mean neck circumference was acquired by averaging together the soft palate neck circumference and thyroid cartilage neck circumference. OSA status was not known at the time of CT reconstruction and examination.

The STOP-BANG questionnaire, an eight-point “yes-or-no” questionnaire used for screening patients to assess and describe one's risk of developing OSA, was translated to the Hebrew language by a certified translator. Topics addressed in the questionnaire include snoring, tiredness, observed apneic events, blood pressure, BMI, age, neck circumference, and gender. Scores of 0 to 2 are considered low risk; 3 to 4 indicates intermediate risk, and 5 of more delineates high risk.^[14]^ The patients whose scans were adequate for reconstruction were contacted telephonically by a single blinded interviewer; STOP-BANG questionnaires were blindly completed within a 2-month period following completion of scan reconstruction and analysis. All patients answering the questionnaire did so voluntarily. Additional information collected included patient age at the time of CT scan, sex, BMI, and smoking status. 3D reconstructions and anthropometric measures were saved on the hospital-wide PACS database, and questionnaires were saved as hard documents and kept in the patient's file.

### 2.4 Statistical analysis

Categorical variables were described as numbers and percentages. Continuous variables were evaluated for normal distribution using histograms and Q–Q plots. Normally distributed continuous variables were reported a mean and standard deviation (SD) and skewed variables were reported as median and interquartile range (IQR). Categorical variables were compared using chi-squared test of Fischer's exact test and continuous variables were compared using ANOVA, independent samples *t*-test, Kruskal–Wallis test, or Mann–Whitney test. Correlation between continuous variables was evaluated using Spearman's rank correlation coefficient. Multinominal logistic regression, which was multivariate, was used to evaluate the association between each of the measurements and the STOP-BANG categories after controlling for age and sex. Chi-squared automatic interaction detection (CHAID)^[15]^ was used to divide each measurement into categories according to the STOP-BANG category status. All significant tests were 2-sided. *P* < .05 were considered statistically significant. SPSS was used for all statistical analysis (IBM SPSS Statistics for Windows, v.23. IBM Corp. 2013, Armonk, NY).

## 3 Results

Of the 660 patients, 23 were under the age of 18 at the time of CT scan and therefore removed from the study. Another 139 scans were not adequate for analysis and also removed from the study. The remaining 498 patient scans were reconstructed and measurements obtained. Of these 498 patients, 37 were unable to be contacted for various reasons (i.e., phone number no longer in service, phone calls not answered, not home during any contact attempts, etc.) and 34 died between CT scan acquisition and contact attempt, making them also unable to be contacted, for a total of 71 lost to follow-up. The remaining 427 patients whose scans were able to be reconstructed and who were successfully contacted were included in the final analysis. Patient characteristics are described in Table 1. Additionally, when comparing those included to the study to those lost to follow-up, there was no significant difference between gender (56% vs. 57.7% male, *P = *0.78), imaging procedure performed (83.1% vs. 81.7% CTA, *P = *0.76), age (58.98 ± 16.77 vs 61.71 ± 18.77, *P = *0.21), or BMI (26.89 ± 4.35 vs 27.05 ± 5.88, *P = *8.36).

**Table 1:**
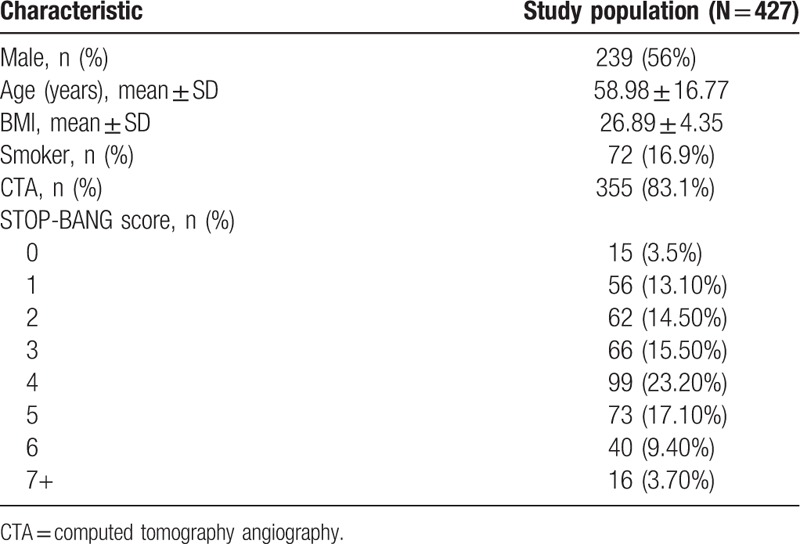
Study population descriptive characteristics.

Patients were also grouped based on STOP-BANG score as low risk (STOP-BANG = 0–2), intermediate risk (STOP-BANG = 3–4), or high risk (STOP-BANG = 5+) for developing OSA. 133 (31.1%) patients fell into the low risk category, 165 (38.6%) into the intermediate risk category, and 129 (30.2%) into the high risk category.

**Figure 2:**
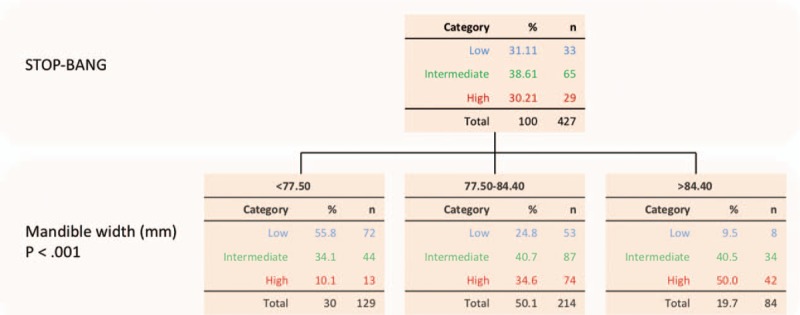
Comparison of STOP-BANG score risk groups to mandible size.

Mandible width mean, SD, and range of the entire population and of each risk group are summarized in Table 2. A moderate positive correlation (*r* = .416, *P* < .001) was seen upon comparison of mandible width to STOP-BANG score. Furthermore, analysis of mandible width compared to STOP-BANG risk classification groups indicated statistically significant differences between each risk group (for low vs. intermediate, intermediate vs. high, and low vs. high risk) (*P < *.001 for all). Controlling for age and sex produced an odds ratio (OR) of 1.065 (confidence interval (CI) = .996–1.140) (*P = *.065) when comparing low versus intermediate groups and an OR of.1.111 (CI = 1.030–1.199) when comparing low versus high risk groups (*P = *.007). Furthermore, separation of mandible size into narrow (<77.50 mm), medium (77.50–84.40 mm), and wide (>84.40 mm) mandibles corresponds to the differences in OSA risk (Fig. 2), with narrow mandible size consisting predominantly of low OSA risk patients, medium mandible size consisting predominantly of intermediate OSA risk patients, and wide mandible size consisting predominantly of high OSA risk patients.

**Table 2:**
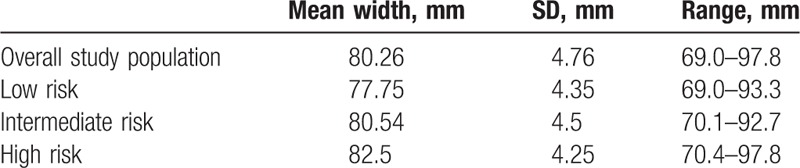
Mandible width statistics.

The correlation between STOP-BANG and various other proposed risk factors, including mandible width, are described in Table 3. Mandible width, NFV, and NFV:AWV ratio all demonstrate moderate associations, whereas neck circumference demonstrates a higher association. Only AWV lacks any association with STOP-BANG. Furthermore, mandible width is more strongly correlated to the risk of developing OSA than is NFV:AWV ratio, although less strongly associated than neck circumference or NFV. At the same time, only mandible width and neck circumference demonstrated significant differences between all risk groups (*P < *.001 for both variables); NFV was significantly different between low to intermediate groups (*P < *.001) and low to high risk groups (*P < *.001), but no significant difference in NFV between intermediate to high risk groups (*P = *.075). NFV:AWV ratio's significance pattern resembled that of NFV alone. There was a significant difference in NFV:AWV ratio between the low to intermediate groups (*P < *.001) and low-to-high risk groups (*P < *.001), but no significant difference in NFV:AWV ratio between intermediate to high risk groups (*P = *.451).

**Table 3:**
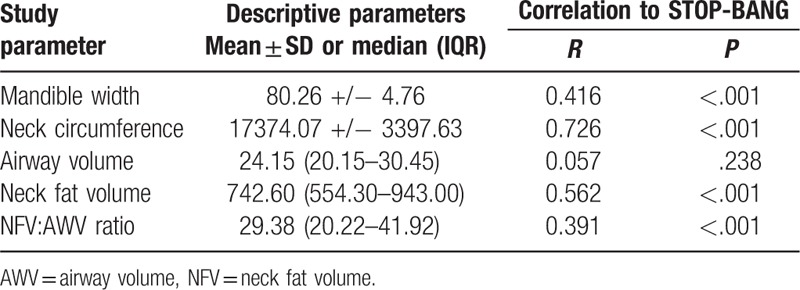
Comparison of associations for various factors.

## 4 Discussion

Identification of OSA risk factors is important to be able to develop a more reliable clinical screening process in order to identify patients that may need further testing and treatment. Craniofacial abnormalities are a class of documented abnormalities, but is a wide category, with some factors being investigated more than others. Results from this study demonstrate that mandible width, which has previously not been researched in regard to OSA, falls into the category of craniofacial abnormalities that pose as risk factors for OSA. Mandible width demonstrates a clear moderate positive correlation with the risk of developing OSA, as seen from comparisons of mandible width to the STOP-BANG survey in the current study.

Several prior studies have been performed to assess various other components, aside from mandible size, on the presence of OSA. Neck obesity has been identified as the primary problem in the development of OSA, with all other factors being secondary to neck obesity.^[16]^ Our study agreed with this assessment through our comparison of NFV to the risk of developing OSA. Building off of this is the idea that neck size influences OSA development, as well, and that variations in neck circumference are closely correlated with OSA.^[16]^ Similarly, larger neck cross-sectional areas are seen in patients with OSA compared to those without OSA,^[17]^ as is in accordance with our observation on neck circumference. However, the correlation between neck circumference and OSA may actually be related to the gender difference and the increased prevalence of OSA in males compared to females due to the fact that males tend to have larger neck circumferences.^[18]^

Davies and Stradding explain that neck size is a predictor of obstruction, which is the primary problem in apneic episodes,^[16]^ thereby indicating a correlation between NFV and AWV. We therefore included NFV:AWV are a parameter in our study and observed a positive correlation. Results of our previous study indicated that NFV:AWV ratio is positively correlated with mortality.^[19]^ The current study builds upon this relationship by demonstrating that NFV:AWV is positively correlated with OSA, and mortality is a long-term sequela of untreated OSA.

The exact role of AWV in OSA is still debated. Smaller AWVs in patients with OSA compared to those without have been observed.^[17]^ Other studies indicate that AWV alone is not a good predictor, but that the volume or variations in volume in particular parts of the airway, such as the retroglossal airway^[16]^ or velopharynx^[20]^ are influential in OSA. Our results do differ on this point, due to our lack of observation of an association between AWV and OSA risk.

While other craniofacial abnormalities have been thoroughly investigated, jaw width seems to be the exception.^[6]^ Chi et al. do suggest increased mandibular width in apneic patients when compared to controls, although their findings did not meet cutoffs for statistical significance.^[9]^ Another study in the Japanese population observed a wider angle of mandibular divergence in OSA patients when compared to controls, although they did not observe any difference between mandibular internal widths.^[21]^ Despite these 2 studies not finding correlations between mandible width and OSA, the use of mandibular advancement devices as an alternative OSA treatment to CPAP does suggest mandibular involvement in the disease pathology.^[20]^ Mandibular advancement devices treatment effect is that the device increases upper airway volume^[22,23]^ and one hypothesis of this mechanism is through displacement of the parapharyngeal fat pads away from the airway and anterior positioning of the base of the tongue muscles.^[22]^ Building off of this proposed mechanism, we wonder if mandible size influences muscle positioning, which could subsequently influence airway size and opening. Although there is no literature to support or refute our hypothesis, this could be an area for future investigation, as well.

Knowledge of the relationship between mandible width and OSA may turn mandible width into an anthropometric tool for clinicians. In the event of a patient undergoing head and/or neck CT scan mandibular width should be measured and documented by the radiologist, and if large mandibular width is noted, general practitioners and sleep specialists will know to screen the patient for OSA. In this case, the patient suffering from undiagnosed OSA will then be diagnosed and treated or if the patient does not have OSA he can still be monitored so that in the event of developing OSA, he is treated promptly, thereby avoiding the long-term sequela of the disease. While imaging allows for a more accurate means of measuring the mandible, many individuals have never undergone head/neck CT scanning from which to obtain mandible size measurements. In this case, even using a tape measure to measure jaw size could give an approximation towards mandible width and allow for screening, diagnosis, and prevention to be applied.

The current study is not without any limitations. As is often the case, there was some loss to follow-up, which is known to cause selection bias. However, our description of the study population and the population lost to follow-up being equivalent minimizes the potentially confounding effect by ruling out come confounding variables such as BMI and age but does not eliminate it entire. Additionally, all the patients included in the study had a co-morbidity that lead to their presentation at our institution and CT performance. While mandible size is a fixed anatomical parameter that does not change with co-morbidities, the co-morbidity could potentially influence participants’ answers to the STOP-BANG questionnaire, which could potentially confound questionnaire scores. For instance, one question in the survey asks about daytime somnolence, but there are several causes for daytime somnolence, with OSA being only one such cause. Similarly, another question in the survey asks about snoring loudly, but sound description is a subjective measure that needs to be reported by other person, not the patient himself. This could have been eliminated by assessing patients with known OSA diagnoses and comparing them to controls without OSA or by using polysomnography in place of the STOP-BANG questionnaire, as it is a more objective measure. However, this was not done because OSA is known to be significantly under-diagnosed and doing so would have significantly limited the sample size of the study. Finally, STOP-BANG is a widely accepted surrogate for OSA risk but an indirect measure of OSA. Therefore, despite the strong, promising results of this study, further research should be continued using apnea-hypopnea index and polysomnography as a measure to confirm and strengthen these findings.

## 5 Conclusion

The current study demonstrates that mandible size displays a moderate positive correlation with the development of OSA, although it is not as strong of a predictor as NFV or neck circumference. Given this association, mandible size may be included in the group of craniofacial abnormalities considered to be risk factors for the development of OSA. Awareness of the association can increase OSA screening, and subsequently increase OSA diagnosis and treatment, thereby preventing future complications of the condition. However, given that this study relies on the STOP-BANG survey as a surrogate for measuring OSA risk, further research should include correlating mandible size to objective OSA diagnosis on polysomnography, which would strengthen the results of the current study.

## Author contributions

**Conceptualization:** Hillel S. Maresky.

**Data curation:** Hillel S. Maresky, Jaron Tepper, Haim Gavriel.

**Formal analysis:** Miriam Michelle Klar, Tomer Ziv Baran.

**Investigation:** Miriam Michelle Klar.

**Methodology:** Hillel S. Maresky.

**Project administration:** Miriam Michelle Klar.

**Resources:** Sigal Tal.

**Supervision:** Miriam Michelle Klar, Sigal Tal.

**Writing – original draft:** Miriam Michelle Klar.

**Writing – review & editing:** Hillel S. Maresky, Jaron Tepper, Haim Gavriel, Tomer Ziv Baran, Colin M. Shapiro, Sigal Tal.

Miriam Michelle Klar orcid: 0000-0001-7609-5470.
